# Effect of moisture content variation on dielectric properties of various plant leaves at microwave frequencies

**DOI:** 10.1038/s41598-024-64266-3

**Published:** 2024-06-08

**Authors:** Pratipal D. Chauhan, Deepak H. Gadani, Vipin A. Rana

**Affiliations:** Department of Physics, University School of Sciences, Gujarat University, Ahmedabad, Gujarat 380009 India

**Keywords:** Plant sciences, Environmental sciences, Materials science, Physics

## Abstract

Complex permittivity of Corn, Jowar, Ashoka and Banana plant leaves was measured using a Vector Network Analyzer (Anritsu Shockline Model-MS46322A) over 500 MHz to 15 GHz frequency range, at different moisture contents. The dielectric constant ($\varepsilon$′) and dielectric loss ($\varepsilon$″) of the leaves decrease with decrease in moisture content over this frequency range. For the leaves having moisture content less than certain critical moisture level (*CML*), the values of $\varepsilon$′ and $\varepsilon$” remain almost constant with frequency variation. $\varepsilon$″ does not increase appreciably with the increase in moisture content up to *CML*. *CML* is found to vary for different types of leaves. At higher moisture level, $\varepsilon$′ and $\varepsilon$″ exhibit frequency dependence. Above certain moisture level in the leaves, $\varepsilon$″ starts to increase slowly with the increase in frequency above 3 GHz approaching towards the dielectric relaxation of water. The values of $\varepsilon$′ and $\varepsilon$″ were also measured for the extract of leaves and compared with the values calculated using Stogryn equations for the same salinity, and the results agree with the calculated values. The measured values of $\varepsilon$′ and $\varepsilon$″ for the leaves were compared with the values calculated using Debye–Cole dual dispersion dielectric model and are found to match very well.

## Introduction

The complex permittivity of a material determines the behaviour of the material under the influence of *EM* (Electric magnetic) field^1^. The dielectric properties of a material are associated with the storage and heat conversion representing dissipation rate of the applied EM field. The knowledge of dielectric properties of the material can be applied in the development of radio and microwave heating technology. The dielectric constant is a key factor in determining a material′s emissivity since it governs how strongly microwaves interact with materials^2^. The complex dielectric constant in a heterogeneous medium, such as soil or plant material, is a sum of all the individual dielectric constants of the constituent parts (e.g., texture structure, water, air, etc.). The dielectric constant is also affected by temperature, moisture content, salinity, and is dependent on microwave frequency of measurement. In the past two decades, monitoring of vegetation canopy using microwave remote sensing technique has attracted good attention. Correlating the important vegetative characteristics, dielectric characteristics, and orientations to the observed data is crucial for this strategy to be successful^3^. Understanding the relationship between the reflecting, refracting, and absorbing (briefly scattering) coefficients of vegetation canopies is essential for the success of *RS* (Remote sensing) applications, resulting in improved understanding and implementation of RS approaches for managing and monitoring vegetation and forest resources^4^. The amount of water in the leaves is a major factor that affects the dielectric properties of the leaves. On the other hand, the changes in the plant dynamics are mainly governed by the moisture content in the leaves. The deficiency of water to a plant leads to the drying of the leaves, due to which the tips and edges of its leaves start to turn brown^5^. Due to deficiency of water supply for a longer period, the plant may die down or its flowers and fruits may not receive enough nutrients, leading to reduction in quality and quantity of crop yield. On the other side, over watering of plants causes plant to die down due to lack of oxygen available to the roots^5^. The main aim of the study is to detect the effect of water content present in the crop leaves on dielectric properties of the leaves. Findings of such study can be helpful in establishing a corelation between dielectric properties with the health of the plants and crop yield. For this purpose, we selected various corps whose plant leaves have varying shapes and sizes. These plants are widely grown in the Gujarat region.

Corn ranks third important crops in India behind rice and wheat in terms of its coverage area and contribution to the production of all food grains. It is grown over all agro-ecological areas contributing to the production of 22 million tons^6^. Being one of the most widely consumed crops in the world, Corn is grown all over the world. Sorghum (Jowar) is grown in about 100 different nations, with 66 of those cultivating more than 1000 ha or producing more than 1000 t of it^7^. In both tropical and temperate climates, Jowar is a significant cropping plant that is utilized to produce food grains. The production and growth of Corn and Jowar can be severely affected by water stress^8^. Ashoka, commonly found in the Himalaya, Kerala, Bengal, and the entire southern part of India, is among India’s most famous and historic trees. Due to Ashoka’s medicinal potential, it has been widely used to treat a variety of diseases. Ashoka is venerable in India and is trustworthy source of medicine that has extensive uses in Ayurveda, Unani, and homoeopathy. It is utilized in various pharmacological activities like those that fight against cancer, menorrhagia, oxytocic, and microbes^9^. The medical benefits of Ashoka leaves are that it is antibacterial, antidiabetic, anthelmintic, *CNS* (Central nervous system) depressant, anti-menorrhagic, uterine tonic, analgesic, anti-inflammatory, anti-ulcer, anti-cancer, larvicidal, and anti-oxytocin^10^. Thus, the study of Ashoka tree leaves at various water contents is important for the estimation of health of the crop. The inexpensive and excellent nutritious content of the banana make it a very popular fruit. In India, it is one of the most significant fruit crops, right after mango. Gujarat is the third-largest producer of bananas, contributing 13.44% of global production. Gujarat state is home to a horticultural crop of banana that requires a lot of water to flourish^11^. Ulaby and Jedlicka suggested a model applicable to Corn and Wheat leaves over 1–20 GHz frequency range by utilizing a number of microwave remote sensing systems for the observations^12^. In this study, complex permittivity of Corn (Zea mays), Jowar (Sorghum bicolor), Ashoka (Saraca asoca) and Banana (Musa acuminate) leaves were measured over 500 MHz–15 GHz frequency interval, and the measured values of complex permittivity were compared with the values calculated using Debye- Cole dual dispersion dielectric model^12^.

### Dielectric measurement

**Figure 1:**
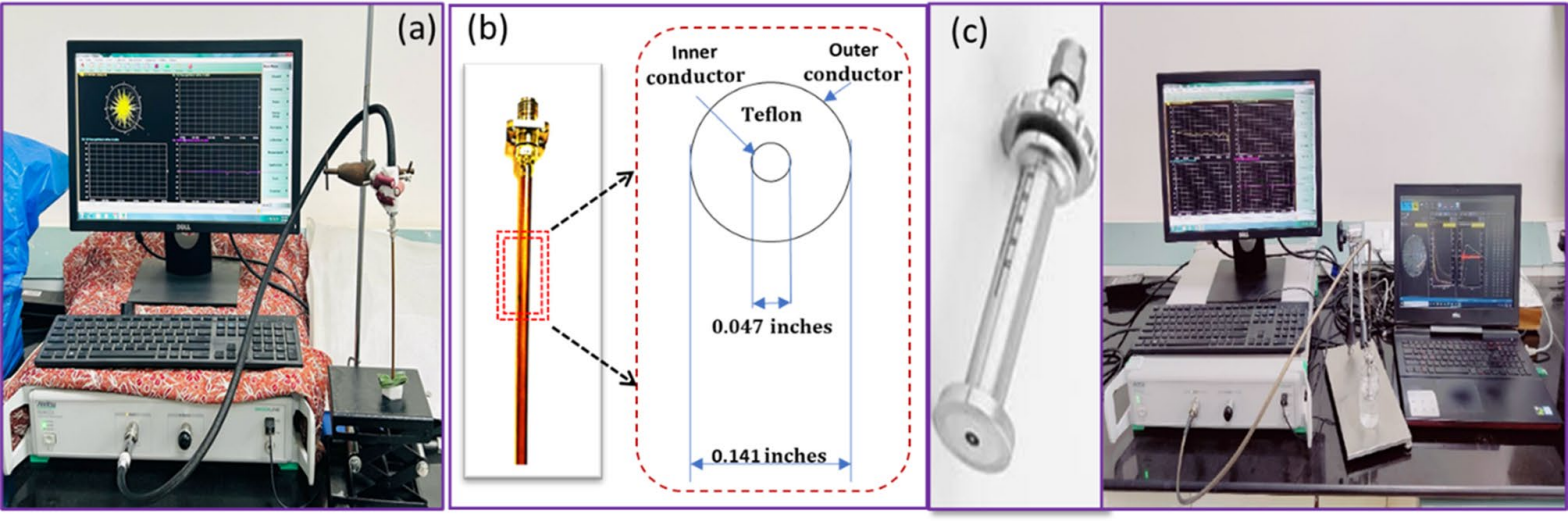
Dielectric measurement setup (**a**) Vector Network Analyzer (Anritsu Shockline Model-MS46322A), (**b**) Semi-rigid coaxial cable (0.141-inch diameter), (**c**) DAK-3.5 probe.

The Vector Network Analyzer (Anritsu Shockline Model-MS46322A) was used for the measurement of complex permittivity of leaves over 500 MHz to 15 GHz. The Vector network analyzer was first calibrated using a calibration kit (T05LK50A) up to the end of a flexible cable. A semi-rigid coaxial cable (0.141-inch diameter, and length 17 cm) without flange and open at one end, was connected at the end of the flexible cable and mated with the Vector Network Analyzer (VNA) Fig. 1a. Now the open end of semirigid coaxial cable (Fig. 1b) was also calibrated by using three standard terminations with Air as open, Mercury as a short, and Acetone (of AR Grade) as a standard liquid, to measure their reflection coefficients. After this, to check the accuracy of measurement the reflection coefficient of Methanol AR-grade (Analytical Research grade) was measured. The reflection coefficient values of three standard terminations and Methanol were used to calculate the values of $\varepsilon$′ and $\varepsilon$″ as explained earlier^13,14^. If the measured values of $\varepsilon$′ and $\varepsilon$″ for Methanol agree within 5% and 10%, respectively with the calculated values using Debye model^15^, the probe could be used for the measurement of $\varepsilon$′ and $\varepsilon$″ for the leaves. About 25 leaves were staked to achieve thickness greater than 3 mm^16^, and measurements were carried out for different moisture contents of the leaves^13,14^. After the measurements the leaves were completely dried by keeping them in oven at 75 °C for 24 h. The same process was repeated for different types of leaves. The gravimetric moisture content (${\text{M}}_{\text{g}}$) was calculated using^17^.

1 $${\text{M}}_{{\text{g}}} = \frac{{{\text{w}}_{{\text{V}}} - {\text{w}}_{{\text{d}}} }}{{{\text{w}}_{{\text{V}}} }}$$

Here, ${\text{w}}_{\text{V}}$ is the weight of wet leaves sample, and ${\text{w}}_{\text{d}}$ is the weight of dry leaves sample.

About 50 ml extract of leaves was obtained using a handheld juicer, which was filtered using a double filter with permeability of 100 micrometer each. The salinity of the extracts of the leaves was measured in terms of TDS (Total Dissolved Salts) in ppm, using a TDS meter. TDS meter HM digital (TDS-3) was used for the measurement of TDS. The measurement of ε′ and ε″ for the leaves’ extract (saline solution) was carried out over the same frequency range. DAK-3.5 probe (shown in Fig. 1c) with frequency range of 200 MHz–20 GHz have been used to carry out the dielectric measurement as explained in our earlier paper^18^*.* It is worth mentioning here that we have used flanged open ended coaxial probe for measurement of complex permittivity of the leaves' extract (saline solution), whereas un-flanged open ended coaxial probe was used for the leave samples. The reason for this distinction is that there is a possibility of creation of air gaps between the flange and leaf surface, while performing the measurements with a flanged probe, which could produce errors in the measured dielectric data. However, in case of probe without a flange, there is no possibility of creation of air gaps between the probe and the leaf surface, and therefore the error is minimized.

The complex permittivity of leaves for various MC at certain microwave frequencies were calculated using^12^:

2 $$\varepsilon_{v} = \varepsilon_{r} + v_{fw} [4.9 + \frac{75.0}{{1 + jf/18}} - j\frac{18\sigma }{f}] + v_{b} [2.9 + \frac{55.0}{{1 + (jf/0.18)^{0.5} }}]$$

3 $$\varepsilon_{r} = 1.7 - 0.74M_{g} + 6.16M_{g}^{2} = {\text{ non}} - {\text{ dispersive residual component}}$$

4 $$v_{fw} = M_{g} (0.55M_{g} - 0.076) = {\text{ volume fraction of free water}}$$

5 $$v_{b} = 4.46M_{g}^{2} /(1 + 7.36M_{g}^{2} )$$

6 $$\sigma = 1.27 = {\text{ ionic conductivity for free water}} \left( {{\text{in}}S/m} \right)$$

where, *Mg*, ${\upvarepsilon }_{\text{v}}$*,*${\text{v}}_{\text{b}}$ *and f* respectively represent the gravimetric moisture content (MC), the complex permittivity of vegetation, and volume portion of bulk vegetation including bound water, and frequency (GHz).

## Results and discussion

**Figure 2:**
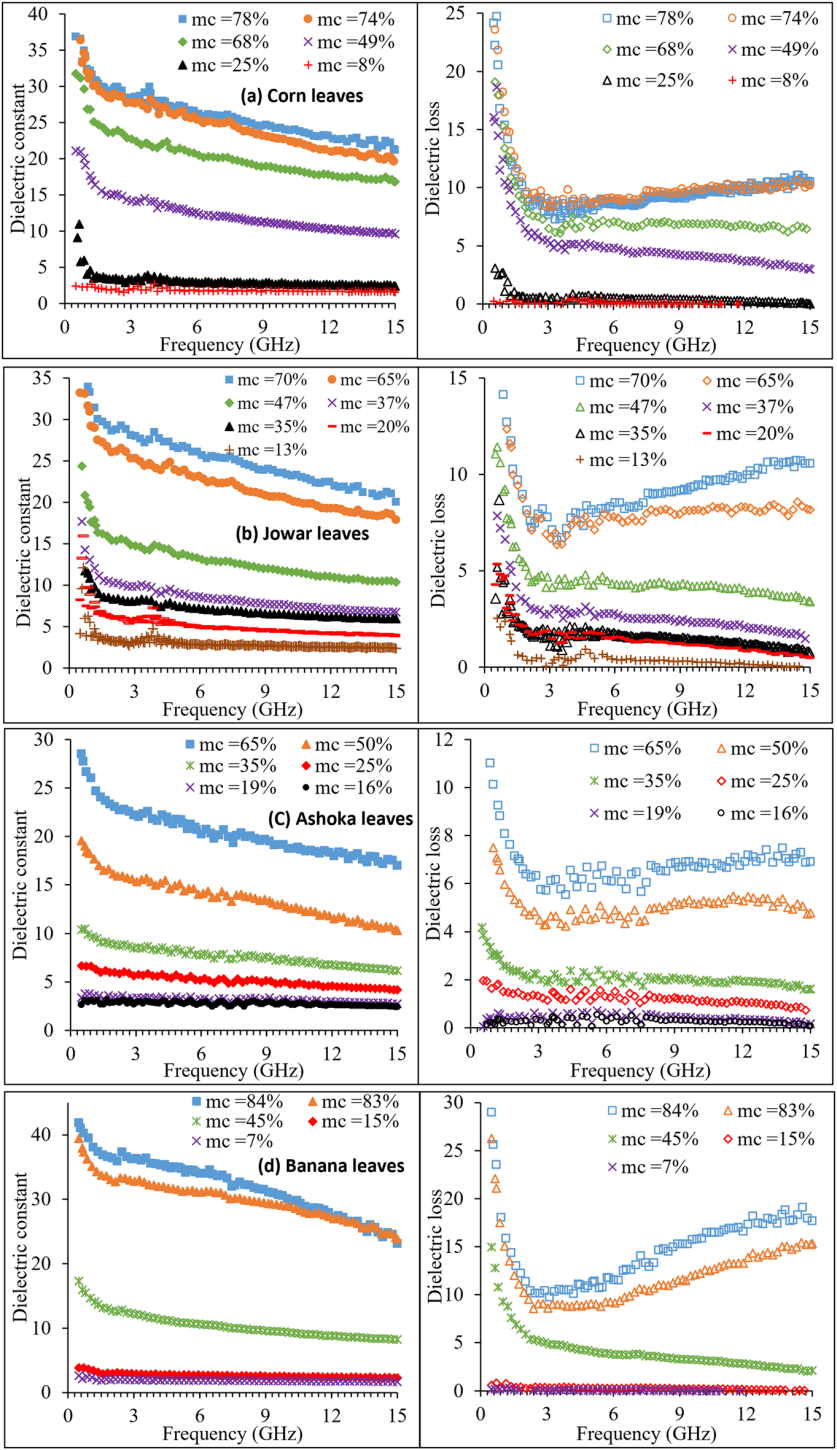
The dielectric constant and dielectric loss of (**a**) Corn, (**b**) Jowar, (**c**) Ashoka, and (**d**) Banana leaves at 0.5 GHz to 15 GHz frequency range.

Figure 2 shows the variation of dielectric constant ($\varepsilon$′) and dielectric loss ($\varepsilon$″) of the (a) Corn, (b) Jowar, (c) Ashoka, and (d) Banana leaves for various moisture contents, over the microwave frequency range. It can be observed that value of $\varepsilon$′ increases with increase in moisture content over the given frequency range. The increase in the value of $\varepsilon$′ with increase in moisture content is due to the enhanced diffusivity, and solubility of ions and various solutes into the bulk leaf matrix, which increases the number of free ions and permanent dipoles contributing to the polarization^19^. The value of $\varepsilon$′ increases as the frequency decreases. For the leaves having higher moisture contents, as frequency decreases below 2 GHz, the dielectric constant increases rapidly. The dielectric constant of the leaves may be considered as a mixture of dielectric constant of bulk vegetation, free water and bound water^12^. Water molecules that can move freely inside the vegetation material represent free water, whereas bound water molecules are tightly bound with the organic compounds of vegetation. Bound water is physically or chemically bound with the cell walls of vegetation leaves, which interact strongly with the neighboring solute molecules of large size^19^. The response of water molecules to an applied electric field is hindered by such physical forces, which is responsible for the increase in relaxation time, and hence decrease in relaxation frequency. The relaxation frequency of free water is 18 GHz at 22 °C, whereas that of the bound water is about 180 MHz^12^, which is responsible for the rapid increase in the dielectric constant of leaves as frequency decreases towards 500 MHz. The dielectric constant of bound water as well as free water decreases with increase in frequency from 300 MHz to 20 GHz^19^, and hence the value of $\varepsilon$′ for the leaves decreases with increase in frequency for all moisture levels.

From Fig. 2a–d it can be observed that the dielectric loss $\varepsilon$″ of drier leaves remains almost constant over the frequency range of measurement. For the leaves having moderate moisture contents, the values of $\varepsilon$″ decrease rapidly with increase in frequency up to about 2 GHz, after that the values of $\varepsilon$″ decrease slowly with the increase in frequency. At lower moisture content in the leaves, most of the water molecules present in the leaf blade such as veins, midrib, and mesophyll are in the bound form. The dielectric loss of bound water is larger than that of the free bulk water and it decreases with the increase in frequency over the microwave frequency region^19^, and hence $\varepsilon$″ for leaves having lower moisture content decreases with the increase in frequency. For the leaves having higher moisture contents, the values of $\varepsilon$″ decrease rapidly with increase in frequency up to about 3 GHz, after that the values of $\varepsilon$″ start to increase with increase in frequency. The rapid increase in the dielectric loss, at lower frequency end, may also be attributed to the ionic loss mechanism due to the presence of large numbers of ionic identities present in the bulk leaf structure^19^. For the leaves having higher moisture contents, the number of free water molecules per unit volume of bulk vegetation material increases with increase in moisture content in the leaves, which is responsible for the increase in the value of $\varepsilon$″ at frequency above 3 GHz, approaching towards the relaxation frequency of free water. Thus, at higher microwave frequencies, the dielectric loss spectra for these high moisture content leaves follow behaviour of free water, regardless of the presence of salinity since the ionic losses become negligible at these frequencies, and the dipolar or relaxation losses become dominant as observed by Shrestha et al.^19^.

### Dielectric properties of leaves’ extract

**Figure 3:**
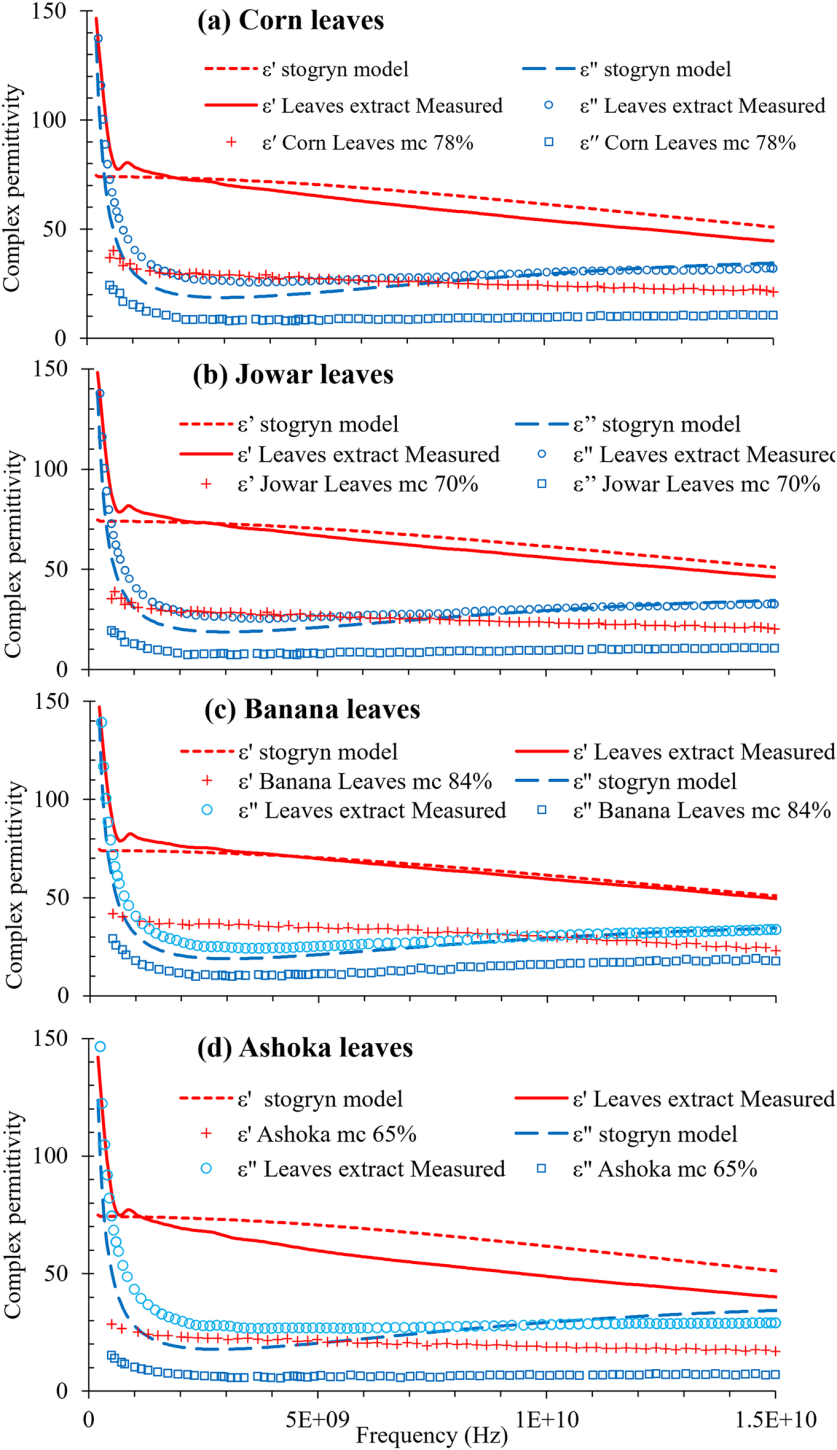
Variation of experimental and calculated values (using Stogryn equations) of $\varepsilon$′ and $\varepsilon$″ with frequency for the (**a**) Corn, (**b**) Jowar, (**c**) Ashoka, and (**d**) Banana leaves and their extract.

The measured amount of TDS for the extract from the (a) Corn, (b) Jowar, (c) Ashoka, and (d) Banana leaves is found to be respectively 8240 ppm, 8320 ppm, 7460 ppm and 8520 ppm. El-Rayes and Ulaby^16^, also found the salinity of fluid extracted from a corn stalk to be 7000 ppm, whereas Shrestha and Wood found the salinity of alfalfa leaves at 52% moisture contents to be 4000 ppm^19^. The verification of the dielectric data of the leaves was carried out by plotting the variation of $\varepsilon$′ and $\varepsilon$″ with frequency for the leaves at their highest moisture contents, along with their extract, and the values calculated using Stogryn equations^21^ corresponding to their respective TDS values as shown in Fig. 3a–d. The variation of $\varepsilon$′ and $\varepsilon$″ for the extract of leaves with that for free water of similar TDS agrees appreciably with each other. The leaf blade consists of many layers such as midrib, veins, cuticle, epidermis, mesophyll, etc. along with air, free saline water and bound water^20^, each having different dielectric properties. Thus, the lower values of $\varepsilon$′ and $\varepsilon$″ for the leaves in comparison with those of free water is due to the presence of bound water along with different components of bulk leaf having different dielectric properties. As per the equations of Stogryn^21^, the value of dielectric loss increases as frequency decreases below 2 GHz for saline water as shown in Fig. 3a–d, at 25 °C temperature. Thus, the rapid increase in the value of $\varepsilon$″ with decrease in frequency below 2 GHz can be associated with the presence of salinity in the vegetation leaves.

**Figure 4:**
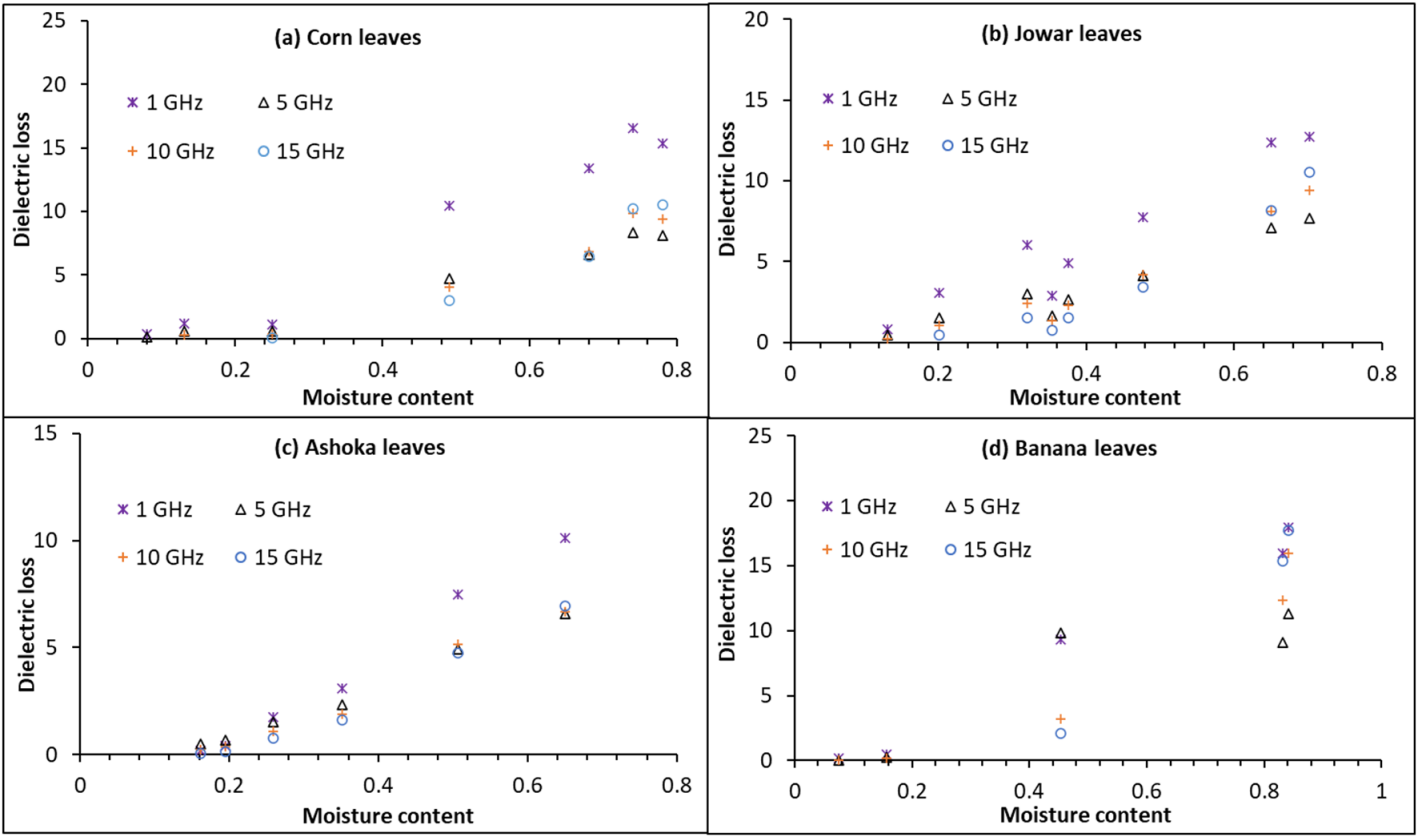
Variation of dielectric loss with moisture content for the leaves of Corn, Jowar, Ashoka, and Banana, at different frequencies.

Figure 4 shows the variation of $\varepsilon$″ with moisture content for the Corn, Jowar, Ashoka, and Banana leaves at four different frequencies. It can be observed that for the Corn, Jowar, Ashoka, and Banana leaves, the value of $\varepsilon$″ does not increase significantly up to 25%, 13%, 19%, and 15% moisture content, respectively. Above these moisture levels the value of $\varepsilon$″ starts to increase appreciably. Similar results were observed by Shrestha et al. ^19^ for alfalfa leaves. The moisture level up to which the variation of $\varepsilon$″ is governed by the bound water molecules is called critical moisture level (*CML*)^19^. Thus, we can say that the value of *CML* for the Corn, Jowar, Ashoka, and Banana leaves, is approximately 25%, 13%, 19%, and 15%, respectively. To estimate exact *CML* for the leaves with better accuracy, more observations at smaller intervals of moisture contents could be taken. Here it can be observed that *CML* for different types of leaves is not the same. As observed the salinity of the leaves under consideration varies around 8000 ppm, and the value of $\varepsilon$″ for the saline water is higher than that for the bound water at these frequencies. For moisture content < *CML*, the water in leaves is only in the bound form ^(19)^, and hence the dielectric loss of the leaves does not increase appreciably. For moisture content > *CML*, both the free and bound water molecules are present in the leaves, and hence the value of $\varepsilon$″ increases.

## Comparison with Debye–Cole model

**Figure 5:**
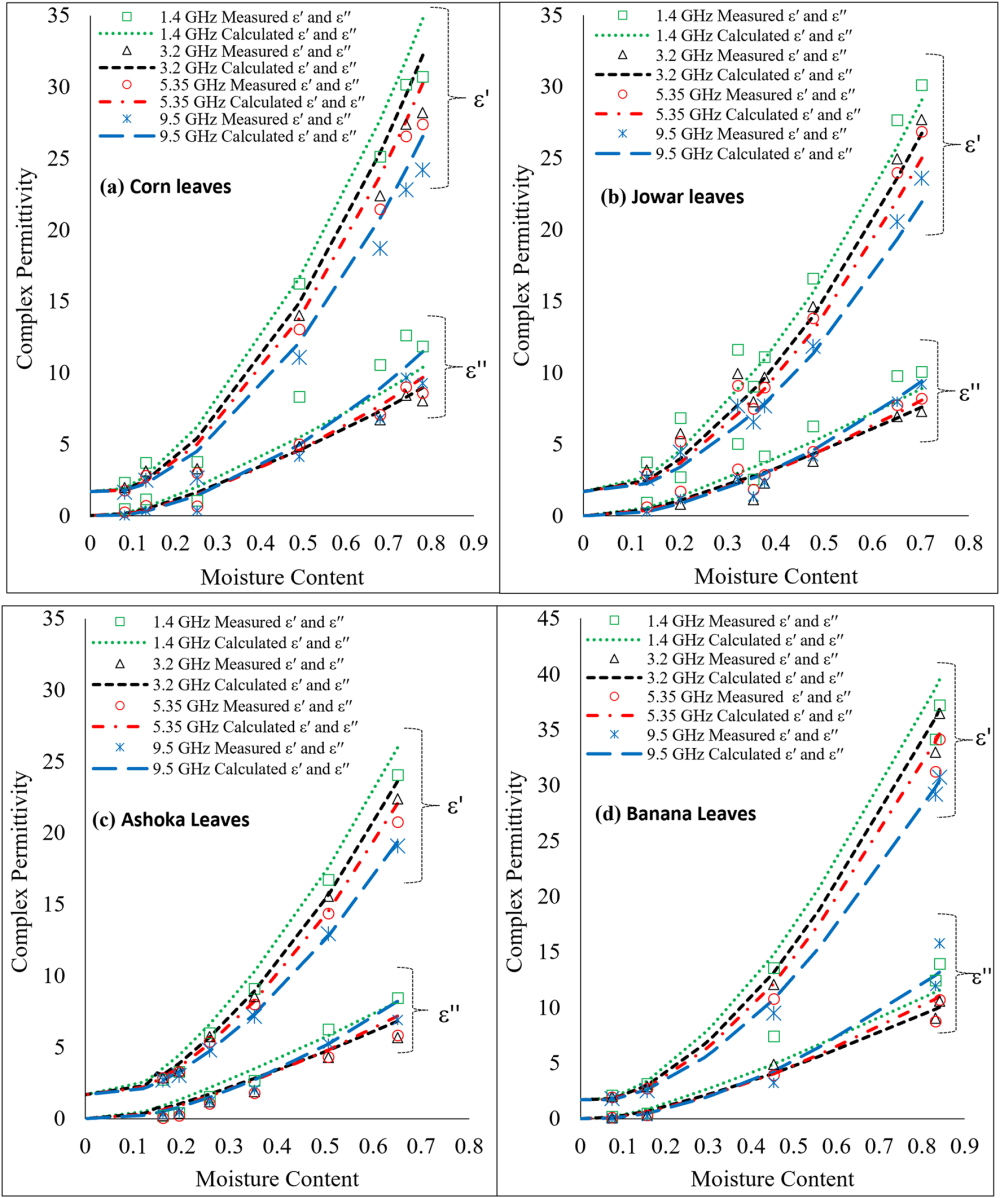
The variation of complex permittivity of the leaves with gravimetric moisture content, at fixed frequencies, compared with the values calculated using the Debye–Cole dual dispersion dielectric model represented by dashed lines.

The measured values of $\varepsilon$′ and $\varepsilon$″ for Corn, Jowar, Ashoka, and Banana leaves at L-Band (1.4 GHz), S-Band (3.2 GHz), C-Band (5.35 GHz) and X-Band (9.5 GHz) were compared with the values calculated using the Debye–Cole dual dispersion dielectric model^12,16^, as a function of gravimetric moisture content. This model considers the presence of bound and free water in vegetation along with other constituents of bulk vegetation. It can be observed from Fig. 5a–d that the values of $\varepsilon$′ and $\varepsilon$″ for the leaves increase rapidly with increase in moisture content at L-band microwave frequency as compared to that at S-band microwave frequency of 3.2 GHz. At lower moisture contents in the leaves, more bound water molecules are present in the leaves, and the values of $\varepsilon$′ and $\varepsilon$″ for bound water increase as frequency decreases from 18 to 0.3 GHz^19^, which plays an important role in increasing of $\varepsilon$′ and $\varepsilon$″ at lower moisture contents in the leaves. At higher moisture contents, the dielectric loss of the leaves increases more rapidly at frequency of 9.5 GHz as compared to that at frequency of 5.35 GHz. At higher moisture contents in the leaves, more free water molecules are present in the bulk leaf samples. This indicates that the presence of free water in leaves can be detected in a better way at 9.5 GHz as compared to that at C-band microwave frequency. Further, it can be observed that for the leaves $\varepsilon$″_*Ashoka*_ < $\varepsilon$″_*Corn*_ < $\varepsilon$″_*Jowar*_ < $\varepsilon$″_*Banana,*_ at 1.4 GHz which is dependent on the TDS level of the water present in the leaves. But at higher frequency of 9.5 GHz and above the values of $\varepsilon$″ depend mainly on the amount of water content in the leaves.

## Conclusion

The complex permittivity of the (a) Corn, (b) Jowar, (c) Ashoka, and (d) Banana leaves was measured over 500 MHz to 15 GHz frequency range. The value of $\varepsilon$′ and $\varepsilon$″ for the leaves is found to increase with increase in moisture content. At moisture content up to *CML* the dielectric behaviour of the leaves is governed by bound water molecules only and hence $\varepsilon$″ does not increase appreciably with the increase in moisture content. At moisture contents above *CML*, $\varepsilon$″ increases with increase in moisture content in the leaves. It has been observed that the *CML* is different for different types of leaves. Further study about the broad band dielectric behavior of vegetation can lead to detect the wetness and health of the vegetation canopies. The measured values of $\varepsilon$′ and $\varepsilon$″ for the extract of leaves were compared with the values calculated using Stogryn equations for water of similar salinity as that of the extract and are found to agree well with each other. The comparison of measured values of $\varepsilon$′ and $\varepsilon$″ for the leaves with the values calculated using Debye–Cole dual dispersion dielectric model is in good agreement. Reported complex permittivity data of the leaves of four significant plants will find its usefulness in monitoring and managing crop yield using remote sensing.

## Data Availability

“The datasets used and/or analyzed during the current study are available from the corresponding author on reasonable request.” *Permission to collect leaves*: We have permission to collect the plant leaves. One of our co-authors Pratipal D. Chauhan is Son of a farmer and the leaves were collected from his own field with consent of his father. Information on the specimen leaves was identified Pratipal D. Chauhan and D. H. Gadani, and the specimens were collected by Pratipal D. Chauhan. After the measurements, the arc of the leaves was extracted to measure its actual salinity and complex permittivity over the same frequency range of measurement as a part of the manuscript. So the leaves are not deposited in a public herbarium nor other public collection providing access to deposited material.
